# Comprehensive Investigation of the Commercial ELP-20 Electron-Beam Lithography Resist

**DOI:** 10.3390/mi17010004

**Published:** 2025-12-19

**Authors:** Meruert Qairat, Aliya Alzhanova, Mustakhim Pshikov, Renata Nemkayeva, Nazim Guseinov, Sergey Zaitsev, Mukhit Muratov

**Affiliations:** 1 National Nanotechnological Laboratory of Open Type, Al-Farabi Kazakh National University, Almaty 050040, Kazakhstan; meruert.kajrat03@gmail.com (M.Q.); mustahim.pshikov@gmail.com (M.P.); quasisensus@mail.ru (R.N.); solar_neo@mail.ru (N.G.); 2 Department of Technical Physics, L.N. Gumilyov Eurasian National University, Astana 010000, Kazakhstan; 3 Institute of Microelectronics Technology and High Purity Materials, Russian Academy of Sciences, Chernogolovka, 142432 Moscow, Russia; bachokg@gmail.com; 4 Kazakh Physical Society, Almaty 050040, Kazakhstan

**Keywords:** ELP-20 resist, spin coating, sensitivity, contrast, electron-beam lithography (EBL)

## Abstract

A systematic experimental study of the positive-tone resist ELP-20 was conducted, covering its structural properties, film-formation behavior, and response to electron-beam exposure. Raman spectroscopy demonstrated the methacrylate nature of the resist and its spectral correspondence to poly(methyl methacrylate) PMMA, which enabled direct comparison both with PMMA itself and with existing methacrylate-based resists. Spin-coated films prepared from 3–11 wt.% solutions exhibited a robust power-law dependence of thickness on spin speed, h_∞_ ∝ ω^−0.48 ± 0.01^, and showed high thickness uniformity. The concentration dependence of the film thickness at a fixed spin speed allowed identification of the polymer–coil overlap region and enabled estimation of the effective molecular weight of the polymer base, *M_eff_* = (25 ± 7) kg/mol. Lithographic characterization indicated a decrease in sensitivity with increasing electron energy, with a sensitivity of approximately 40 μC/cm^2^ at 25 keV. A depth-dependent dose-distribution model provided an energy-independent average contrast value of *γ* ≈ 1.67. The results present a coherent and systematic description of ELP-20 behavior under electron-beam exposure and establish a basis for its further use in lithographic processing.

## 1. Introduction

Electron-beam lithography (EBL) remains one of the most precise technologies for micro- and nanostructuring, enabling the fabrication of structures with sub-10 nm resolution [1]. The combination of high spatial resolution, maskless patterning capability, and a wide range of adjustable process parameters, including precise modulation of the exposure dose during patterning, provides the method with substantial process flexibility. As a result, EBL is widely used for producing functional structures in micro- and nanoelectronics [2,3], photonics [4,5], and plasmonics [6], where a binary exposure mode has traditionally been employed. The development of the technique has led to grayscale electron-beam lithography (g-EBL), which relies on precise local dose modulation. This approach significantly extends the capabilities of conventional electron lithography, enabling the accurate fabrication of three-dimensional micro- and optical structures of complex topology [7]. The performance of EBL is largely determined by the properties of the resist, which represents a key element of the process and defines the achievable sensitivity, contrast, resolution, resistance to subsequent technological operations and pattern fidelity. The most widely used resist materials include positive-tone methacrylate-based resists and negative-tone inorganic resists. Among commercially available resists, poly(methyl methacrylate) PMMA with a molecular weight of 950 K (950 kDa) is considered the benchmark positive-tone material. As a representative of the methacrylate resist family, it enables the formation of highly uniform films and supports electron-beam patterning with resolution below 10 nm [1,8]. Its sensitivity, however, remains relatively low and typically lies within ~80–400 μC/cm^2^ at incident electron energies of 10–30 keV [1,8,9,10] and ~300–700 μC/cm^2^ at 100 keV [8,11,12,13], whereas its contrast remains moderate *γ* ≈ 2–4, with a possible increase up to ~7–10 under low-temperature development [14]. An additional limitation is the relatively low plasma-etch resistance, which further constrains its technological applicability [10]. Modern commercial methacrylate-based resists, including methacrylate copolymers such as ZEP 520 and the chemically semi-amplified resist CSAR 62, as well as the SML resist, offer a more favorable combination of sensitivity, contrast, resolution, and plasma resistance. At incident electron energies of 10–100 keV, ZEP 520 and SML typically exhibit dose requirements of ~10–600 μC/cm^2^, depending on processing conditions. ZEP 520 shows higher sensitivity, developing at the lower end of this dose range, while both resists provide high lithographic contrast *γ* ≈ 6–12 and maintain resolution of ~5–15 nm [10,15,16]. For CSAR 62, the sensitivity achieved at 30–100 keV is ~55–170 μC/cm^2^, while the contrast varies within *γ* ≈ 5–14 depending on the process conditions, and the resist simultaneously provides sub-10 nm resolution along with enhanced plasma resistance [8,17,18]. Among inorganic negative-tone resists, hydrogen silsesquioxane (HSQ) holds a special position, providing sub-10 nm resolution. At incident electron energies of 10–100 keV, HSQ exhibits a relatively low sensitivity of ~0.7–3 mC/cm^2^, while its typical contrast values are *γ* ≈ 3–4. The use of salt-modified alkaline developers enables an increase in contrast up to *γ* ≈ 12, which significantly improves the fidelity of densely packed nanostructures. Combined with its high resistance to plasma etching, HSQ remains a key negative-tone material for high-resolution EBL [19,20,21].

Furthermore, experimental studies show that the lithographic performance of a resist is determined not only by its intrinsic resist properties but also by the choice of the “resist–solvent” and “resist–developer” systems, as well as by the accompanying process parameters. Film thickness and uniformity are governed by spin-coating conditions, the nature of the solvent, and the degree of residual solvent removal during thermal processing. Together, these factors determine the quality of the polymer film and influence its lithographic behavior [22,23,24]. Exposure conditions, including the incident electron energies, define the total absorbed energy and its distribution throughout the resist thickness, directly affecting the lithographic characteristics [10,16,25]. The development conditions and the choice of developer determine the dissolution kinetics and also have a significant impact on the key lithographic properties of the resist [1,8,14,26]. The combined action of these factors determines the effective values of sensitivity, contrast, and resolution, highlighting that process optimization is equally important as the resist selection. Modern resist materials, when considered together with all the factors mentioned above, possess a broad experimental basis and allow achieving an optimal balance between sensitivity, contrast, resolution, and process reproducibility. Nevertheless, selecting the most appropriate resist for a specific task remains a decisive factor for attaining the required characteristics.

In addition to the widely used resists discussed above, the commercial Russian positive-tone material ELP-20, developed by JSC “NIOPIK” (Moscow, Russia) specifically for EBL applications, is of considerable interest. Despite its commercial availability and established use in practical technologies [27], the scientific literature provides no data on its molecular composition or effective molecular weight. Furthermore, no studies have been published on its film-formation behavior under spin-coating conditions, and systematic investigations of its lithographic performance, including sensitivity and contrast, are likewise unavailable. The absence of such information complicates a direct comparison of ELP-20 with established commercial resists and limits its applicability in tasks requiring quantitative control of the lithographic process. The aim of this work is to experimentally determine the structural and fundamental lithographic characteristics of the positive-tone resist ELP-20, including its molecular composition, effective molecular weight, spin-coating kinetics, and dose–response parameters such as sensitivity and contrast under moderate-energy electron exposure (≤30 keV). The use of well-established processing conditions typical of methacrylate family resists provides reliable calibration and enables an objective assessment of the potential of ELP-20 as a material suitable for research-oriented and technological lithography applications.

## 2. Materials and Methods

Standard monocrystalline silicon wafers with a diameter of 100 mm and a thickness of 500 µm were used as substrates for spin-coating of the resist. The wafers were cut into ~25 × 25 mm^2^ square pieces by diamond scribing. Prior to resist deposition, the substrates underwent sequential ultrasonic cleaning: first in acetone (purity ≥ 99.99%) for 10 min, followed by isopropyl alcohol (purity ≥ 99.8%) for another 10 min. Subsequently, the samples were dried on a hot plate at 200 °C for 5 min to remove residual moisture. The commercial ELP-20 positive resist was purchased from NIOPIK (Moscow, Russia). Solutions with mass concentrations of 1, 2, 3, 4.8, 6, 7.2, 8 and 9.5 wt.% were prepared from the initial 11 wt.% stock solution by dilution in chlorobenzene (purity 99.9%) under thorough stirring. Concentration control was performed using gravimetric determination of the solid residue on an analytical balance Sartorius CPA225D (Sartorius AG, Göttingen, Germany). Spin-coating was carried out with a SCK-200 spin coater (Instras Scientific, Ridgefield Park, NJ, USA). The process consisted of a preliminary spreading stage at 500 rpm for 5 s, followed by the main spin step at 1500–6000 rpm for 60 s. This procedure enabled evaluation of the film thickness dependence on both spin speed and solution concentration. After deposition, the samples were baked on a 5-inch magnetic hot plate (FOUR E’S SCIENTIFIC, Guangzhou, China) at 180 °C for 3 min to remove residual solvent and densify the films. For Raman spectroscopy studies, a commercial positive PMMA (poly(methyl methacrylate)) resist with a molecular weight of 950 K was employed as a reference in the form of a 4 wt.% solution in anisole MicroChem (Kayaku Advanced Materials, Westborough, MA, USA). Electron-beam exposure was performed on a Quanta 3D 200 i DualBeam scanning electron microscope (FEI Company, Hillsboro, OR, USA) equipped with the NanoMaker hardware–software system (Interface, Moscow, Russia), which provided pattern generation and exposure data preparation. A test pattern in the form of a dose wedge was used to characterize the lithographic response of the resist. The wedge consisted of a linear array of rectangles sized 20 × 4 µm^2^, exposed at linearly increasing doses. Electron-beam exposures were carried out at energies of 5, 15, and 25 keV, while maintaining a constant beam current of 30 pA in all experiments. The dose ranges were 0–10 µC/cm^2^ (step 0.5 µC/cm^2^), 0–30 µC/cm^2^ (step 1.5 µC/cm^2^), and 0–50 µC/cm^2^ (step 2.5 µC/cm^2^) for 5, 15 and 25 keV, respectively. Development of the exposed samples was carried out in a standard developer system consisting of methyl isobutyl ketone (MIBK, purity ≥ 99.0%) and isopropyl alcohol (IPA, purity ≥ 99.8%) mixed in a 1:3 (*v*/*v*) ratio. Development time was 20 s at room temperature (25 °C). The process was completed by rinsing in deionized water for 5–10 s, followed by drying under a nitrogen stream. Surface topography, film thickness, and developed resist profiles were characterized using an atomic force microscope Solver Spectrum (NT-MDT, Zelenograd, Russia) operated in tapping mode at a scan rate of 0.1 Hz, ensuring high vertical resolution. To determine the initial film thickness after spin-coating, a mechanical scratch was made through the resist layer using metal tweezers, and an AFM scan was performed across the resist-substrate step. Cantilevers NSG01 (TipsNano, Tallinn, Estonia) with a tip radius < 10 nm, nominal stiffness 1.45–15.1 N/m, and resonance frequency ~170 kHz were used. Data processing was carried out in the Nova Px 3.2.5 software package, enabling extraction of height profiles and quantitative analysis of fabricated structures. The measured wedge profiles were converted into an exposure dose scale for further sensitivity and contrast evaluation. The molecular structure of ELP-20 and PMMA 950 K was studied by Raman spectroscopy using a Solver Spectrum system (NT-MDT, Zelenograd, Russia) equipped with a 473 nm excitation laser of ~0.8 mW power. Focusing was achieved with a 100× objective lens. A 600/600 diffraction grating was employed, providing a spectral resolution of 4 cm^−1^, with an acquisition time of 200 s. All experiments were conducted in controlled-cleanliness laboratory facilities, although not certified to ISO cleanroom standards.

## 3. Results and Discussion

### 3.1. Raman Spectroscopy of ELP-20 Resist

**Figure 1:**
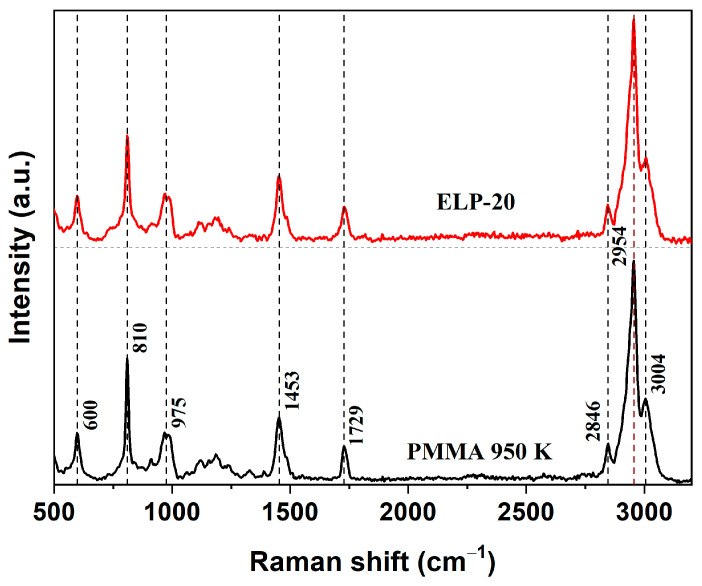
Raman spectra of ELP-20 and PMMA 950 K resists obtained under identical experimental conditions (excitation at 473 nm).

Raman spectroscopy was employed to analyze the molecular structure of the ELP-20 resist and to compare it with the widely used positive resist PMMA 950 K. Both materials were spin-coated on quartz substrates, forming films with a thickness above 1 µm. Figure 1 shows the spectra, which exhibit a characteristic set of vibrational bands in the range of 500–3200 cm^−1^ for both samples.

In the low-frequency region, the band at 600 cm^−1^ corresponds to C–C–O vibrations, and the band near 810 cm^−1^ is assigned to C–O–C vibrations, both typical for methacrylate esters [28,29,30,31,32,33,34]. The peak at 975 cm^−1^ originates from CH_3_ rocking modes, while the signal at 1453 cm^−1^ is attributed to C–H deformations of α-methyl groups [28,29,30,31,32,33,34]. The characteristic band at 1729 cm^−1^ is assigned to the C=O stretching vibration and is a well-established fingerprint feature of methacrylate polymer structures [29,30,31,32,33,34]. In the high-frequency region, the bands at 2846, 2954, and 3004 cm^−1^ are associated with symmetric and asymmetric C–H stretching in methyl and methylene fragments of the side chains. The band near 2846 cm^−1^ primarily corresponds to symmetric CH_2_ stretching, whereas the strong band at 2954 cm^−1^ results from overlapping symmetric and asymmetric CH_3_ vibrations, which dominate the Raman spectrum of PMMA. The weaker feature near 3004 cm^−1^ is usually attributed to overtone or combination modes involving C–H stretching in O–CH_3_ and α-CH_3_ groups [29,30,31,34]. The close correspondence in the positions of the characteristic bands and their relative intensities in the Raman spectra of ELP-20 and PMMA 950 K indicates the identity of the polymer backbone and the presence of the same set of vibrational modes characteristic of poly(methyl methacrylate). This spectral agreement convincingly confirms that the polymer base of the ELP-20 resist is formed by repeating poly(methyl methacrylate) units. At the same time, it should be emphasized that this conclusion is strictly limited to the nature of the polymer backbone and does not imply equivalence of macromolecular characteristics, nor does it preclude the presence of formulation-specific additives introduced at low concentrations that fall below the sensitivity limits of Raman spectroscopy and therefore do not manifest themselves in the recorded spectra.

### 3.2. Investigation of ELP-20 Resist Thin Film Formation by Spin-Coating

**Figure 2:**
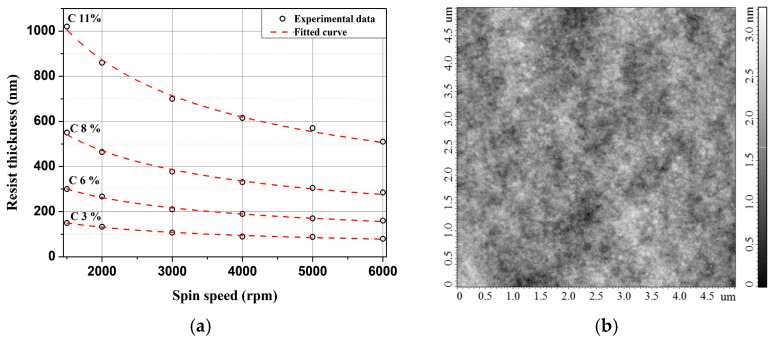
(**a**) Thickness of ELP-20 resist as a function of spin-coating speed for different solution concentrations. (**b**) AFM image of the spin-coated film with a thickness of ~200 nm, prepared at 4000 rpm and baked at 180 °C for 3 min.

The next stage of the study focused on investigating the formation of thin ELP-20 resist films using the spin-coating technique. Film thickness measurements were performed using atomic force microscopy (AFM). Initially, the dependence of the final film thickness on the spin speed was examined. Experimental results demonstrated that within the 1500–6000 rpm range and for solution concentrations of 3, 6, 8 and 11 wt.% the resulting film thickness decreases monotonically with increasing spin speed (Figure 2a). The obtained data are well described by a power-law relationship:

(1) $$h\propto \omega^{-b}$$

where *h* is the film thickness and *ω* is the spin speed. The power-law exponent extracted from fitting the experimental data was *b* = 0.48 ± 0.01.

The dependence of the resist film thickness on the spin speed can be interpreted within the classical theoretical description of spin-coating, which considers the balance between centrifugal and viscous forces together with solvent evaporation. The first model describing the thinning of a Newtonian liquid during spin-coating was proposed by Emslie et al. [35], who neglected solvent evaporation and therefore could not account for the finite residual thickness observed at sufficiently long spinning times. Later, Meyerhofer [36] modified the model [35] by introducing a constant solvent evaporation rate, which led to an expression describing the thinning of a Newtonian liquid under spin-coating conditions:

(2) $$-\frac{dh}{dt}=\frac{2\rho \omega^{2}}{3\eta }h^{3}+E$$

where *η* is the solution viscosity, *ρ* is the solution density and *E* is the solvent evaporation rate. In Ref. [36], solvent evaporation was taken into account under the assumption of a constant evaporation rate. This enabled the introduction of the quasi-steady-state condition, defining the final residual film thickness corresponding to an infinitely long spinning time. Using a two-stage model (viscous and evaporative regimes), Meyerhofer derived an analytical expression that relates this final thickness to the solution parameters and processing conditions:

(3) h∞~ ω−23E13η13c

where *c* is the solution concentration. According to the results reported in Refs. [37,38], the solvent evaporation rate during spin-coating follows the dependence *E* ∝ *ω*^1/2^. Taking this into account, the two-stage model reduces to the universal scaling relation:

(4) h∞~ ω−12η13c

In Equation (4), which describes the dependence of the final film thickness on the spin speed (*h*_∞_ ∝ *ω*^−*b*^), the theoretical exponent is *b* = 0.5. In the present study, the experimental value of this exponent for the ELP-20 resist, obtained by fitting the dependence of the final film thickness on the spin speed under fixed processing conditions, was *b* = 0.48 ± 0.01. This result is in good agreement with the theoretical prediction as well as with previously reported data for PMMA [39,40] and other polymer systems [41,42], indicating that the film-formation process is controlled, among other factors, by solvent evaporation. Deviations of the exponent b from the theoretical value of 0.5 can be attributed to the specifics of solvent evaporation [43], the non-Newtonian behavior of polymer solutions (i.e., shear-dependent viscosity) and the contribution of inertial effects at elevated spin speeds [44]. Furthermore, for all solution concentrations studied the variation in film thickness along the substrate radius from the center to the edge did not exceed 2%, confirming the uniformity of the film thickness. A representative AFM topography image of an ELP-20 film (Figure 2b) demonstrates a smooth surface without noticeable defects. The root-mean-square surface roughness for all samples was below 1 nm, indicating high surface quality and uniformity.

**Figure 3:**
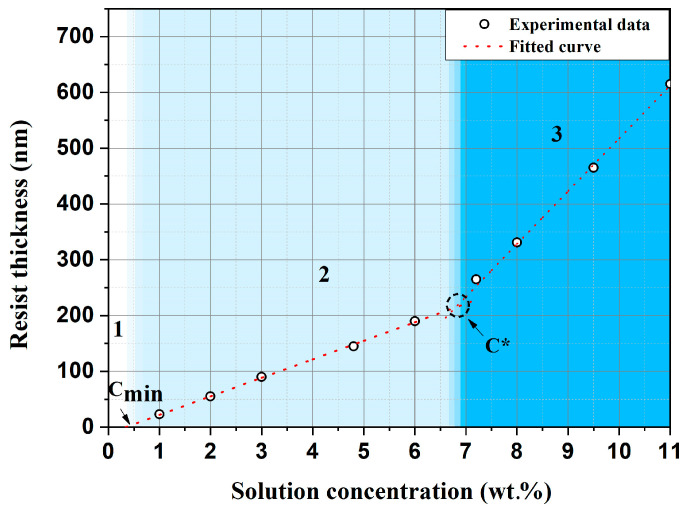
Thickness of ELP-20 resist as a function of solution concentration at a fixed spin speed of 4000 rpm.

To further analyze the influence of processing parameters on film formation, the dependence of the ELP-20 resist film thickness on solution concentration was investigated at a fixed spin speed of 4000 rpm. The selected speed corresponds to typical spin-coating conditions and ensures uniform and reproducible film formation. For a more detailed quantitative assessment, additional solutions with concentrations of 1, 2, 4.8, 7.2, and 9.5 wt.% were prepared. As shown in Figure 3, the film thickness increases with increasing solution concentration. Three distinct concentration regions (labeled 1, 2, and 3 in Figure 3) can be identified, each corresponding to different regimes of film formation and reflecting changes in the rheological and dynamic properties of the solution. This behavior is consistent with current understanding of spin-coating processes in polymer systems [45,46].

To interpret the obtained *h*_∞_(c) dependences, a semi-empirical approach proposed by Schubert [45] was employed. This model represents a modification of the classical Emslie [35] and Meyerhofer [36] descriptions, taking into account the increase in solution viscosity during spin-coating due to solvent evaporation and the associated rise in solution concentration. At low solution concentrations the solution viscosity can be approximated using the Huggins equation: *η* ≈ *η*_0_(1 + [*η*]*c*), where *η*_0_ is the solvent viscosity and [*η*] is the intrinsic viscosity of the polymer in the given solvent. Incorporating viscosity evolution into the film-formation process Ref. [45] yields the following general expression for the final film thickness:

(5) h∞~ ω−12[η]13c

The intrinsic viscosity of the polymer is described by the Mark–Houwink–Sakurada equation, [*η*] = *KM^a^*, where *K* and *a* depend on the «polymer–solvent–temperature» system. For PMMA in chlorobenzene at 25 °C, a value of *a* = 0.72 was used, which is characteristic of a good solvent and taken from the literature [47]. With this consideration, Equation (5) takes the following form:

(6) $$h_{\infty }\sim \;\omega^{-1/2}M^{0.24}c$$

Analysis of the experimental dependence of the ELP-20 resist film thickness on solution concentration (Figure 3) showed that, when a parameter corresponding to the minimum solution concentration *cₘᵢₙ* is introduced into Equation (6), the model satisfactorily describes the experimental data in the range of moderate solution concentrations (region 2 in Figure 3), up to the critical concentration c*. Taking into account the minimum solution concentration, Equation (5) can be rewritten as follows:

(7) $$h_{\infty }\sim \omega^{-1/2}M^{0.24}(c-c_{min})$$

Physically, the parameter *cₘᵢₙ* represents the minimum amount of polymer in the solution required to form a continuous film. Its value obtained by fitting the model to the experimental data is ≈3.9 mg/mL (taking into account the density of chlorobenzene of 1.11 g/cm^3^ at 25 °C) which is comparable to experimental results reported for the “polystyrene–toluene” system where the minimum solution concentration is approximately 2.56 mg/mL [45].

A characteristic transition from a weakly sloped linear region to a steeper increase in film thickness with growing solution concentration (Figure 3, transition from curve 2 to curve 3) indicates the onset of the critical concentration c*, corresponding to the overlap threshold of polymer coils. At *c* < c*, macromolecules exist as isolated chains (dilute regime), whereas at *c* > c* the solution enters the semidilute regime with first weak (unentangled) and then strong (entangled) coil overlap, leading to a pronounced increase in viscosity. According to the statistical coil model c* is reached when the solution concentration inside the volume occupied by a single macromolecule becomes equal to the concentration of polymer in the solution. Thus, the value of c*, identified from the characteristic change in the slope of the film-thickness dependence on solution concentration in the local transition region, was subsequently used to estimate the polymer molecular weight. In the classical framework the overlap concentration c* is defined through the intrinsic viscosity [*η*], treating macromolecules as impenetrable hydrodynamic volumes. In this approximation, the overlap of macromolecular coils is described by the universal criterion [48,49]:

(8) $$c^{*}[\eta ]≅1$$

which reflects the direct relationship between the critical overlap concentration and the intrinsic viscosity of the solution. The experimentally established Mark–Houwink–Sakurada parameters for PMMA in θ- and good solvents were used [47,50,51]. Based on these data, together with the experimentally determined critical concentration c* = 6.8 wt.% (taking into account the chlorobenzene density of 1.11 g/cm^3^ at 25 °C), the effective molecular weight of the polymer base of the resist was estimated. The resulting value *M_eff_* = (25 ± 7) kg/mol for the ELP-20 resist reflects the uncertainty inherent to indirect determination: first by identifying c* and then converting it to molecular weight. A comparable level of uncertainty is reported in the literature for PMMA in θ- and good solvents obtained using various methods, which confirms the validity and reproducibility of the adopted approach. Considering the polydisperse nature of resist materials, this value should be interpreted as an effective molecular weight. For comparison, the molecular weights of commercial PMMA-950 K, ZEP-520, CSAR 62 are ~950, ~57 kg/mol, and ~38 kg/mol, respectively.

With a further increase in solution concentration beyond the critical value c* the solution enters the semidilute regime where substantial overlap and entanglement of macromolecular chains occurs already at the early stages of spin-coating. Under these conditions, the contribution of viscosity becomes dominant; it increases sharply with solution concentration, which restricts radial flow of the solution and results in a higher residual film thickness. In this concentration regime, the experimental data are well described by the semi-empirical linear relationship proposed in Ref. [45]:

(9) $$h_{\infty }\sim S(c-c_{0})$$

where *S* and $c_{0}$ exhibit power-law scaling with molecular weight: *S* ∝ *M*^0.2^, $c_{0}$ ∝ *M*^−0.42^. It should be noted that this model was originally developed for the «polystyrene–toluene» system, where toluene is a good solvent for polystyrene, similar to chlorobenzene being a good solvent for PMMA. Both polymer–solvent pairs are characterized by similar interaction physics, coil conformations, and viscosity evolution when transitioning from the dilute to the semidilute regime. Therefore, application of this model to the PMMA–chlorobenzene system is appropriate and physically justified. Thus, the overall dependence of the final film thickness *h_∞_* on spin speed (rpm) and solution concentration (mass fraction) for the ELP-20 resist can be represented as a piecewise-linear approximation, capturing the transition between different concentration regimes of the solution. The effective molecular weight *M_eff_* (kg/mol) is introduced into the analytical expression in a parametric form. In the dilute regime (*c* < c*, region 2 in Figure 3), the dependence is described by the following expression:

(10) $$h_{\infty 2}\left(\omega ,c\right)=(6.6\cdot 10^{3}\;nm)\cdot {\left(\frac{2000\;rpm}{\omega }\right)}^{0.48}{\left(\frac{M_{eff}}{100\;kg\cdot {mol}^{-1}}\right)}^{0.24}\left(c-0.0035\right)$$

whereas in the semidilute regime (*c* > c*, region 3 in Figure 3), the dependence is described by the following expression:

(11) $$h_{\infty 3}\left(\omega ,c\right)=\left(1.8\cdot 10^{4}\;nm\right)\cdot {\left(\frac{2000\;rpm}{\omega }\right)}^{0.48}{\left(\frac{M_{eff}}{100\;kg\cdot {mol}^{-1}}\right)}^{0.2}\left(c-0.026\cdot {\left(\frac{M_{eff}}{100\;kg\cdot {mol}^{-1}}\right)}^{-0.42}\right)$$

### 3.3. Sensitivity and Contrast of the ELP-20 Resist Under Electron Beam Exposure

**Figure 4:**
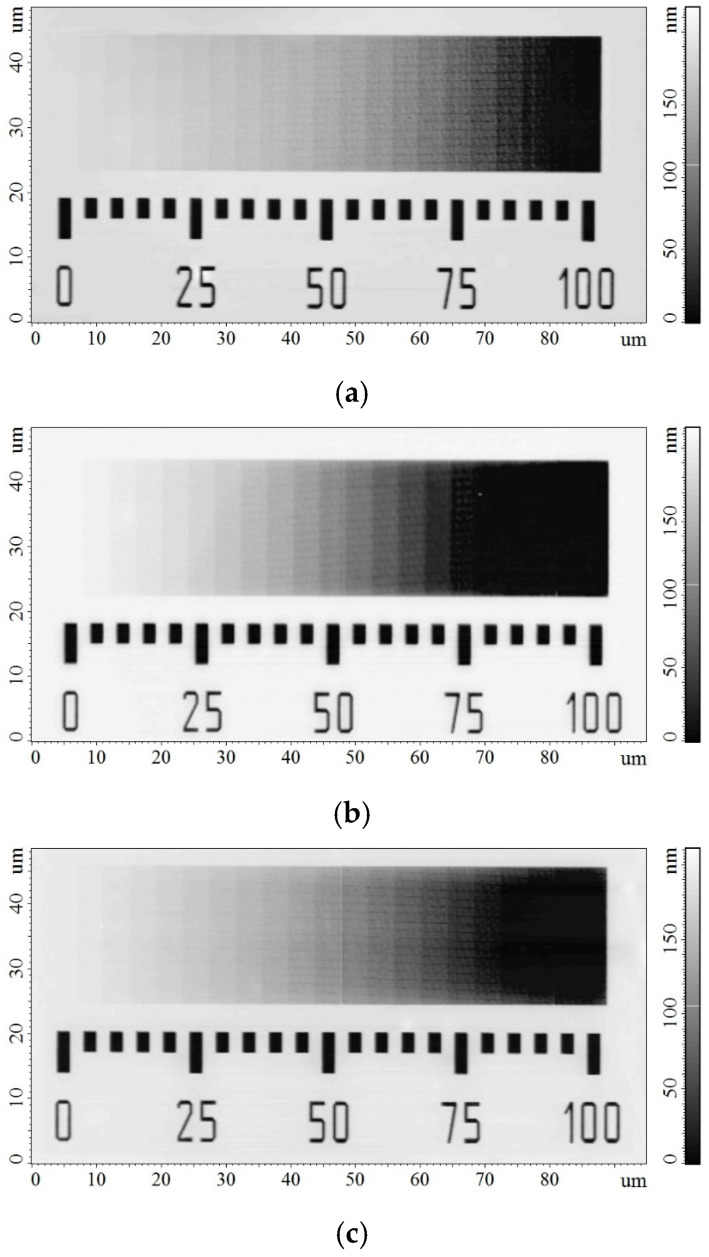
AFM images of dose wedges on the ELP-20 resist after development, corresponding to electron energies of (**a**) 5 keV, (**b**) 15 keV, and (**c**) 25 keV.

Quantitative evaluation of the dose characteristics of the ELP-20 resist was carried out using a dose-wedge test structure consisting of an array of rectangular cells exposed at sequentially increasing doses. This approach enables the formation of a controlled dose gradient along a single coordinate and allows determination of the resist sensitivity and contrast within a single exposure field [52]. A linearly increasing dose gradient was employed in this study. Figure 4 presents AFM images of the developed dose wedges in the ELP-20 resist. Bright regions correspond to unexposed or undeveloped resist, dark regions represent areas that are fully developed and cleared to the substrate, and intermediate gray levels indicate gradual variations in the residual film thickness. Three dose wedges were patterned on a single substrate and exposed at electron energies of 5, 15, and 25 keV with dose ranges of 0–10, 0–30, and 0–50 µC/cm^2^, respectively, corresponding to a full (100%) dose scale for each case. Placing all dose wedges on the same substrate ensured identical exposure and development conditions.

**Figure 5:**
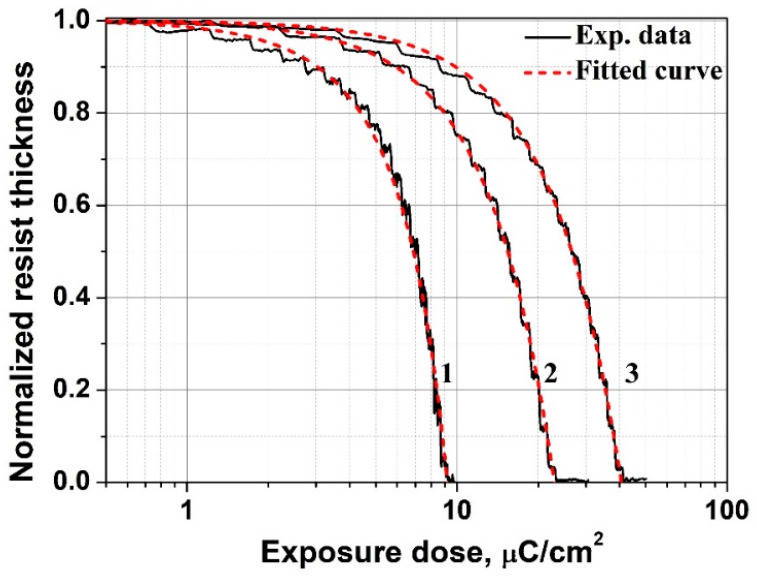
Experimental dependence of the residual thickness of the ELP-20 resist on the exposure dose at electron energies of 5 keV, 15 keV, and 25 keV, denoted by curves (1), (2), and (3), respectively. The corresponding dashed lines (1–3) show the parametric fits of the experimental data obtained using the analytical model (Equation (15)).

Figure 5 shows the averaged dose profiles of the residual film thickness of the ELP-20 resist at electron energies of 5, 15, and 25 keV. These profiles were obtained from the AFM images (Figure 4) by linearly converting the lateral coordinate into the corresponding exposure dose. The initial resist thickness was *h*_0_ = 200 nm, and the residual film thickness is plotted in normalized units, while the dose axis is presented on a logarithmic scale. As seen from the data, increasing the electron energy results in a shift in the dose required to fully develop the resist down to the substrate (sensitivity) toward higher values. This trend reflects the reduced energy deposition density at higher electron energies, which is determined by the energy loss mechanisms described by the Bethe equation [53]. In the electron energy range of 5–25 keV and for a sufficiently thin resist layer, the specific energy loss of electrons in the material can be considered inversely proportional to the electron energy *dE*/*dx* ∝ 1/*E*. Under this assumption, the dose required to fully develop the resist down to the substrate increases proportionally with energy *D*_0_ ∝ *E*. This explains the experimentally observed quasi-linear increase in *D*_0_ with electron energy: at higher energies, electrons lose less energy per unit path length and penetrate deeper into the resist, which requires a higher dose to achieve an equivalent level of exposure compared to low-energy irradiation. The corresponding values of *D*_0_ for different electron energies are summarized in Table 1. It is worth noting that at an electron energy of 25 keV, the ELP-20 resist exhibits a significantly higher sensitivity of approximately 40 µC/cm^2^ compared to PMMA 950 K, for which the sensitivity typically ranges from ~80 to 400 µC/cm^2^ depending on the exposure and development conditions [1,8,9,10]. Moreover, at an electron energy of 5 keV and for a 200 nm thick resist layer, the sensitivity of the ELP-20 reaches values below 10 µC/cm^2^, which is of considerable interest in terms of increasing lithography throughput and may be beneficial for a broad range of relevant technological applications.

To quantify the contrast, the classical model [52,54] was applied in which the contrast is defined by the slope of the linear region of the dose response curve (in logarithmic scale). In this model, the normalized residual resist thickness *h* as a function of the exposure dose *D* is expressed as:

(12) $$\frac{h}{h_{0}}=1-{\left(\frac{D}{D_{0}}\right)}^{\gamma }$$

where *h*_0_ is the initial resist thickness, *D*_0_ is the dose required to fully develop the resist down to the substrate (sensitivity), and *γ* is the contrast. The parameter γ* represents the sharpness of the transition and is defined as the tangent of the slope angle θ* of the dose response curve in the coordinates *h*/*h*_0_ vs. log_10_(*D/D*_0_), in the point *D*_0_ and is expressed as:

(13) γ∗=tgθ∗=dhh0d log10DD0

The contrast *γ* is related to the parameter γ* by the expression *γ* = γ*/ln(10) and the calculated values of the effective contrast *γ_eff_* are summarized in Table 1 for the dose response curves shown in Figure 5 obtained at different electron energies. As seen from the data, the value of *γ_eff_* increases as the electron energy decreases. It should be noted that the classical procedure for determining contrast from the slope of the linear region of the dose response curve in logarithmic scale is based on the assumption of a uniform energy deposition through the resist thickness, under which the dissolution rate follows the *D^γ^* dependence [52,54,55]. In practice, however, at lower electron energies such as 5 keV, the dose distribution becomes increasingly non-uniform, which leads to an overestimation of the contrast value. For this reason, the contrast determined using the classical method [52,54] reflects the influence of the non-uniform energy deposition through the resist thickness and is therefore regarded as the effective contrast. To eliminate the influence of electron energy and substrate type in quantitative contrast evaluation, a model accounting for the depth distribution of deposited energy was applied. This approach enables determination of the intrinsic contrast *γ* independent of exposure conditions. According to the model proposed in [25], the depth-dependent energy deposition in the resist can be written as *D*(*z*) = *D_e_*(1 + *az*) where *D_e_* is the surface exposure dose and the parameter *a* depends on the electron energy and substrate properties. With this non-uniformity taken into account, the classical model describing the resist thickness as a function of exposure dose is written as:

(14) $$\int_{0}^{h\left(D_{e}\right)}\frac{dz}{(1+az{)}^{\gamma }}=const{\left(D_{e}\right)}^{\gamma }$$

Accordingly, the normalized residual resist thickness *H*(*D_e_*) is expressed as a function of the exposure dose under the condition *H*(*D*_0_) = 0 as:

(15) $$H\left(D_{e}\right)=1-\frac{h\left(D_{e}\right)}{h_{0}}$$

In this case, the contrast *γ* and the parameter *a* were determined by numerically fitting the theoretical dependence (15) to the experimental data *H_exp_*(*D*_e_) shown in Figure 5. The fitting parameters were the contrast *γ* and the parameter *a*. The approximation was performed individually for each electron energy and in all cases the coefficient of determination *R*^2^ exceeded 0.97. The results of fitting the model curve to the experimental data of residual thickness versus dose for different electron energies are summarized in Table 1. As can be seen, calculations based on the considered model give an average contrast value of *γ* = 1.67. Furthermore, with increasing energy of the incident electrons, the contrast value calculated using the classical approach gradually approaches the limiting value obtained from the model, whereas the model-derived value itself remains nearly constant over the entire investigated energy range. The obtained contrast value lies within the range typical of PMMA resist under comparable development conditions [8,14,17] (considering that in the present work the contrast was calculated using natural logarithms, whereas a number of studies employ base-10 logarithms [8]).

The behavior of the parameter *a*, which characterizes the gradient of the deposited energy, deserves particular attention (see Table 1). Its sharp decrease with increasing electron energy (from 3.55 × 10^−3^ nm^−1^ at 5 keV to 0.10 × 10^−3^ nm^−1^ at 25 keV) reflects a physically justified reduction in the non-uniformity of the energy profile. This trend is consistent with Monte Carlo simulation results [56,57] and indicates an increase in the deposited energy density from the surface toward the substrate, which is especially pronounced at lower electron energies. For the case of 5 keV and a resist thickness *h*_0_ = 200 nm with a gradient coefficient *a* = 3.55 × 10^−3^ nm^−1^, the product a*h*_0_ ≈ 0.71 indicates a noticeable dose increase through the film thickness. In this case, the ratio of the deposited energy at the surface (*z* = 0) and at the substrate interface (*z* = *h*_0_) is 1.71. In contrast, at 25 keV, the value *ah*_0_ ≈ 0.02 indicates only a weak depth-dependent nonuniformity, meaning that the dose distribution across the resist thickness is close to uniform.

## 4. Conclusions

This study provides a comprehensive evaluation of the positive-tone resist ELP-20, focusing on the parameters that determine its lithographic performance. Raman spectroscopy demonstrated the methacrylate nature of the material and its correspondence to PMMA spectra, which enabled reliable comparison with well-established methacrylate family resists. The spin-coating behavior was examined over a concentration range of 3–11 wt.%, revealing a consistent power-law dependence of film thickness on spin speed, *h*_∞_ ∝ *ω*^−0.48 ± 0.01^. The resulting films exhibited high thickness uniformity and high surface quality (RMS roughness < 1 nm), which are essential for stable lithographic processing. In addition, analytical relations describing the final film thickness as a function of spin speed and solution concentration for different concentration regimes were obtained from the experimental data, and such dependencies have significant practical and technological relevance. The concentration dependence of the film thickness at a fixed spin speed allowed identification of the polymer–coil overlap region and enabled estimation of the effective molecular weight of the polymer base, *M_eff_* = (25 ± 7) kg/mol. Lithographic measurements showed a sensitivity of ~40 μC/cm^2^ at 25 keV and below 10 μC/cm^2^ at 5 keV, consistent with the range typical of high-sensitivity methacrylate-based resists. Application of a depth-dependent dose-distribution model yielded an energy-independent contrast value of *γ* ≈ 1.67, characteristic of PMMA under comparable development conditions. Overall, the results show that ELP-20 exhibits spin-coating behavior characteristic of methacrylate-based resists, shows relatively high sensitivity under moderate-energy electron exposure, and demonstrates stable dose–response characteristics. All lithographic parameters were obtained under processing conditions standard for methacrylate-based resists, confirming the compatibility of ELP-20 with conventional electron-beam lithography workflows.

## Figures and Tables

**Table 1:** Lithographic parameters of the ELP-20 resist determined as a function of electron energy. For comparison, the effective contrast values *γ_eff_* calculated using the classical model and the contrast values *γ* obtained using the model proposed in [25] are also presented.

Electron Energy (in keV)	*D*_0_, µC/cm^2^	*γ_eff_*	*γ*	*a*, (10^−3^ nm^−1^)
5	9.1	2.61	1.69	3.55
15	22.8	1.85	1.65	0.45
25	41.2	1.65	1.67	0.10

## Data Availability

All data is included in the manuscript.
